# ER Stress–Related Genes EIF2AK3, HSPA5, and DDIT3 Polymorphisms are Associated With Risk of Lung Cancer

**DOI:** 10.3389/fgene.2022.938787

**Published:** 2022-07-14

**Authors:** Yongshi Liu, Xiaohua Liang, Hongpei Zhang, Jiajia Dong, Yan Zhang, Juan Wang, Chunmei Li, Xiangbing Xin, Yan Li

**Affiliations:** ^1^ Department of Thoracic Surgery, Tangdu Hospital, The Fourth Military Medical University, Xi’an, China; ^2^ Department of Respiratory Medicine, Tangdu Hospital, The Fourth Military Medical University, Xi’an, China

**Keywords:** lung cancer, gene polymorphisms, single-nucleotide polymorphisms, endoplasmic reticulum stress, case–control study

## Abstract

**Objective:** This study aimed to evaluate the associations between endoplasmic reticulum (ER) stress–related genes *EIF2AK3/*PERK, *HSPA5/*GRP78, and *DDIT3/*CHOP polymorphisms and the risk of lung cancer.

**Methods:** Six single-nucleotide polymorphisms (SNPs) of *EIF2AK3*, *HSPA5*, and *DDIT3* were genotyped in 620 cases and 620 controls using a MassARRAY platform.

**Results:** The minor allele A of rs6750998 was a protective allele against the risk of lung cancer (*p* < 0.001), while the minor alleles of rs867529, rs391957, and rs697221 were all risk alleles that may lead to multiplied risk of the disease (r*p*_rs867529_ = 0.002; *p*_rs391957_ = 0.015; *p*_rs697221_ < 0.001). Moreover, the rs6750998-TA/AA genotypes were protective genotypes against the risk of lung cancer (*p* = 0.005); however, the rs867529-GC/CC, rs391957-CC, and rs697221-GA/AA genotypes were associated with elevated lung cancer risk (*p*_rs867529_ = 0.003, *p*_rs391957_ = 0.028, and *p*_rs697221_ = 0.0001). In addition, *EIF2AK3*-rs6750998 was associated with a decreased risk of lung cancer under dominant, recessive, and log-additive models (*p* < 0.05). By contrast, the *EIF2AK3*-rs867529 was correlated with an increased risk of the disease under dominant and log-additive models (*p* = 0.001). Moreover, *HSPA5-*rs391957 was related to an elevated risk of the disease under recessive and log-additive models (*p* < 0.02). *DDIT3*-rs697221 was identified to have a significant association with the risk of lung cancer under all three genetic models (*p* < 0.01).

**Conclusion:** Our results provide new insights on the role of the ER stress–related genes *EIF2AK3*, *HSPA5*, and *DDIT3* polymorphisms for lung cancer risk.

## Introduction

At present, lung cancer is still a malignant tumor with the highest morbidity and mortality worldwide and is one of the biggest enemies that threaten human health (Siegel et al., 2021; Yang et al., 2022). The World Health Organization statistics show that there were approximately 2.2 million new cases and 1.8 million death cases of lung cancer in 2020, the incidence and mortality rates are 11.4% and 18.0%, respectively (Mattiuzzi and Lippi, 2020). In China, due to increasing air pollution in the process of industrialized urbanization, the highest prevalence of tobacco use, the gradual arrival of an aging society, and changes in lifestyles, the incidence and mortality rates of lung cancer have been on the rise (Cao and Chen, 2019). The treatment of lung cancer mainly includes surgical resection, chemotherapy, radiotherapy, and molecular targeting drugs (Hirsch et al., 2017). Although the treatment of lung cancer has made certain progress in recent years, the overall survival is still dissatisfactory (Patel and Weiss, 2020). Most patients progress to the late stage of this disease at diagnosis and miss the best time and means of treatment, leading to a low five-year survival rate. Therefore, it is extremely important to find biomarkers that can be used in the early diagnosis of lung cancer.

The endoplasmic reticulum (ER) is an organelle that is in charge of the synthesis, processing, and modification of protein and thus plays a pivotal role in maintaining proteostasis. When the cells lack nutrition and have low oxygen, calcium imbalance, or oxidative stress, the unfolded and misfolded protein could accumulate, resulting in ER stress (Walter and Ron, 2011). Accumulating evidence have shown that ER stress is deeply involved in the growth, survival, and differentiation of tumor cells (Chen and Cubillos-Ruiz, 2021). PRKR-like ER kinase (PERK) is one of the main stress sensors that mediates ER stress. Generally, PERK is bound to the molecular partner protein causing glucose-regulated protein 78 (GRP78) to become inactive. Under ER stress, the unfolded or misfolded protein is bound to GRP78 competitively, resulting in the dissociation of GRP78 to PERK and activation of the downstream signaling pathway (Volmer et al., 2013). Moreover, activated PERK phosphorylates elF2α and upregulates ATF4 and CHOP, resulting in the activation of a number of genes involved in the biosynthesis and transport of amino acids and intracellular autophagy (B'Chir et al., 2013; Han et al., 2013). Therefore, PERK, GRP78, and CHOP are important proteins involved in ER stress. Previous studies have reported the crucial functions of these genes in the occurrence and metastasis of several types of cancer (Xie et al., 2015; Xu et al., 2019; Zhang et al., 2019). However, little information is found about the single-nucleotide polymorphisms (SNPs) in *EIF2AK3/*PERK, *HSPA5/*GRP78, and *DDIT3/*CHOP in cancer patients, especially those with lung cancer.

In the present study, a total of six candidate SNPs in *EIF2AK3*, *HSPA5*, and *DDIT3* were chosen from previous association studies. The rs6750998 and rs17037621 are intron SNPs in *EIF2AK3* and associated with insulin resistance, high BMI, and the risk of prediabetes (Feng et al., 2014). The rs867529 is a nonsynonymous SNP in *EIF2AK3* and is correlated with the risk of prediabetes and lower bone mineral density (Liu et al., 2012). Moreover, rs17840761 and rs391957 are promoter SNPs in *HSPA5* and have been investigated in patients with gastric and colorectal cancer (Winder et al., 2011). Additionally, rs697221 is a nonsynonymous SNP in *DDIT3* and has been detected in patients with melanoma in a Brazilian population (Francisco et al., 2013). None of these SNPs have been genotyped in patients with lung cancer. Therefore, we genotyped these candidate SNPs in a case–control cohort with 620 lung cancer patients and 620 healthy controls and evaluated these associations with the risk of lung cancer.

## Materials and Methods

### Participants

In this study, 620 lung cancer patients and 620 healthy controls were recruited at the Tangdu Hospital. The diagnosis of lung cancer was established by histopathological examination of biopsy or resected tissue specimens. The patients who had received chemo- or radiotherapy were excluded. The healthy controls were enrolled from cancer-free individuals from the same hospital and were matched to the cases in gender and age. We obtained written informed consent from all subjects. The study was approved by the Ethics Committee of Tangdu Hospital and carried out in accordance with the World Medical Association Declaration of Helsinki—Ethical Principles for Medical Research Involving Human Subjects.

### Genotyping

Six tag SNPs in *EIF2AK3*, *HSPA5*, and *DDIT3* were selected in the present study; these SNPs were with minor allele frequencies (MAFs) >5% in the East Asian populations of 1000 Genomes. The DNA was extracted from the blood samples using the QIAamp DNA Blood Midi Kit (QIAGEN, Germany). The primers were designed using the SEQUENOM MassARRAY Assay Designer 3.0 software. SNP genotyping was performed by SEQUENOM MassARRAY RS1000 (Sequenom Inc., San Diego, CA). The primers used for this study are listed in the Supplementary Material.

### Statistical Analysis

Statistical analyses were performed with SPSS 21.0 statistical package (SPSS, Chicago, IL, United States). The allele frequencies in the cases and controls were tested for departure from the Hardy–Weinberg equilibrium (HWE). HaploReg v4.1 (https://pubsbroadinstituteorg/mammals/haploreg/haploregphp) was used to predict the potential functions of the SNPs. Differences in the demographic variables and allele frequencies between the cases and controls were evaluated using chi-square tests and Welch’s t-tests. Associations between the genotypes and lung cancer risk were evaluated by unconditional logistic regression analysis and expressed by odds ratios (ORs) and 95% confidence intervals (CIs) using SNPstats (https://www.snpstats.net/start.htm). The interaction between SNPs was analyzed by using multifactor dimensionality reduction (MDR) software. The statistical significance was established when *p* < 0.05.

## Results

The basic information of the participants is listed in Table 1. The case group included 384 males and 236 females, and 381 smokers and 239 nonsmokers, with a mean age of 57.09 years; the control group included 381 males and 239 females, and 378 smokers and 242 nonsmokers, with a mean age of 56.61 years. No significant difference was observed in the distribution of sex, age, or smoking status between the two groups (*p* > 0.05). The case group consisted of 294 adenocarcinoma patients, 188 squamous cell carcinoma patients, 113 small-cell lung cancer patients, and 25 other types of lung cancer patients.

**TABLE 1:** Basic information of the participants.

Characteristics	Case (n = 620)	Control (n = 620)	χ2/t	*p*
Gender (%)			0.031	0.860
Male	384 (61.9)	381 (61.5)		
Female	236 (38.1)	239 (38.5)		
Age			0.282	0.563
Mean ± SD	57.09 ± 10.41	56.61 ± 10.64		
Smoking (%)			0.031	0.860
Yes	381 (61.5)	378 (61.0)		
No	239 (38.5)	242 (39.0)		
Pathological types				
Adenocarcinoma	294 (47.4)			
Squamous cell carcinoma	188 (30.3)			
Small-cell lung cancer	113 (18.2)			
Others	25 (4.1)			

The basic information for the candidate SNPs is presented in Table 2. The predicted function according to the HaploReg database showed that rs6750998 and rs17037621 in *EIF2AK3*, and rs17840761 and rs391957 in *HSPA5* were involved in the regulation of the promoter or enhancer histone, changed motifs, and eQTL hits. Moreover, *EIF2AK3*-rs867529 and *DDIT3*-rs697221 were missense variants and led to changed amino acids.

**TABLE 2:** Basic information and predicted functions of candidate SNPs.

SNP	Gene	Position	Allele	Role	Predicted functions
rs6750998	EIF2AK3/PERK	chr2:88583424	T > A	Intron	Motifs changed and eQTL hits
rs17037621	EIF2AK3/PERK	chr2:88606202	T > A	Intron	Promoter/enhancer histone mark, motifs changed, and eQTL hits
rs867529	EIF2AK3/PERK	chr2:88613755	G > C	Missense variant	Ser136Cys
rs17840761	HSPA5/GRP78	chr9:125241700	G > A	Promoter	Promoter histone mark, motifs changed, and eQTL hits
rs391957	HSPA5/GRP78	chr9:125241745	T > C	Promoter	Promoter histone mark, motifs changed, and eQTL hits
rs697221	DDIT3/CHOP	chr12:57517377	G > A	Missense variant	Phe33Leu

SNP, single-nucleotide polymorphism; eQTL, expression quantitative trait locus.

The genotyping call rate in our study was 100%. The MAFs of SNPs in cases and controls are described in Table 3. All of the SNPs were consistent with HWE (*p* > 0.05). By comparing the MAFs of SNPs between the case and control groups, we found that the minor allele A of rs6750998 was a protective allele against the risk of lung cancer (OR = 0.733, 95% CI: 0.609–0.882, *p* < 0.001), while the minor alleles of rs867529, rs391957, and rs697221 were all risk alleles that may lead to the multiplied risk of the disease (rs867529: OR = 1.301, 95% CI: 1.105–1.531, *p* = 0.002; rs391957: OR = 1.256, 95% CI: 1.045–1.510, *p* = 0.015; rs697221: OR = 1.504, 95% CI: 1.234–1.834, *p* < 0.001).

**TABLE 3:** The MAF and HWE of candidate SNPs between lung cancer cases and healthy controls.

SNP	Gene	MAF-cases	MAF-controls	HWE *p*-cases	HWE *p*-controls	OR (95% CI)	*p*
rs6750998	EIF2AK3/PERK	0.21	0.27	0.55	0.61	0.733 (0.609–0.882)	<0.001*
rs17037621	EIF2AK3/PERK	0.34	0.33	0.21	0.99	1.075 (0.910–1.271)	0.394
rs867529	EIF2AK3/PERK	0.40	0.34	0.13	0.93	1.301 (1.105–1.531)	0.002*
rs17840761	HSPA5/GRP78	0.43	0.41	0.74	0.56	1.083 (0.923–1.270)	0.328
rs391957	HSPA5/GRP78	0.26	0.22	0.18	0.99	1.256 (1.045–1.510)	0.015*
rs697221	DDIT3/CHOP	0.23	0.17	0.82	0.32	1.504 (1.234–1.834)	<0.001*

*p < 0.05 indicates statistical significance.

SNP, single-nucleotide polymorphism; MAF, minor allele frequency; HWE, Hardy–Weinberg equilibrium.

The genotype frequencies of SNPs in the cases and controls are shown in Table 4. The wild genotype of each SNP was considered as the reference genotype, and the OR and 95% CI of the heterozygous and homozygous mutational genotypes were evaluated. The results showed that the TA and AA genotypes of rs6750998 were protective genotypes that were associated against the risk of lung cancer (*p* = 0.005); however, the rs867529-GC/CC, rs391957-CC, and rs697221-GA/AA genotypes were all risk genotypes that associated with different levels of elevated lung cancer risk (*p*_rs867529_ = 0.003, *p*_rs391957_ = 0.028, *p*_rs697221_ = 0.0001).

**TABLE 4:** Genotype frequency distributions between lung cancer cases and healthy controls.

SNP	Genotype	Control	Case	OR (95%CI)	*p*
rs6750998	TT	335 (54%)	388 (62.6%)	1	0.005*
	TA	238 (38.4%)	202 (32.6%)	0.73 (0.58–0.93)	
	AA	47 (7.6%)	30 (4.8%)	0.55 (0.34–0.89)	
rs17037621	TT	282 (45.5%)	261 (42.1%)	1	0.440
	TA	272 (43.9%)	294 (47.4%)	1.17 (0.92–1.48)	
	AA	66 (10.7%)	65 (10.5%)	1.06 (0.73–1.56)	
rs867529	GG	268 (43.2%)	212 (34.2%)	1	0.003*
	GC	281 (45.3%)	317 (51.1%)	1.44 (1.13–1.83)	
	CC	71 (11.4%)	91 (14.7%)	1.64 (1.14–2.35)	
rs17840761	GG	222 (35.8%)	202 (32.6%)	1	0.490
	GA	292 (47.1%)	308 (49.7%)	1.16 (0.90–1.49)	
	AA	106 (17.1%)	110 (17.7%)	1.14 (0.82–1.58)	
rs391957	TT	374 (60.3%)	342 (55.2%)	1	0.028*
	TC	216 (34.8%)	228 (36.8%)	1.16 (0.91–1.47)	
	CC	30 (4.8%)	50 (8.1%)	1.86 (1.15–2.99)	
rs697221	GG	424 (68.4%)	364 (58.7%)	1	0.0001*
	GA	182 (29.4%)	221 (35.6%)	1.42 (1.11–1.81)	
	AA	14 (2.3%)	35 (5.7%)	2.92 (1.54–5.51)	

*p < 0.05 indicates statistical significance.

SNP, single-nucleotide polymorphism; OR, odds ratio; CI, confidence interval.

Based on the comparison results of allele and genotype, we further assessed the associations between these SNPs and lung cancer risk under three genetic models (Table 5). We found that *EIF2AK3*-rs6750998 polymorphism was associated with a decreased risk of lung cancer under dominant, recessive, and log-additive models (*p* < 0.05). By contrast, the *EIF2AK3*-rs867529 was correlated with an increased risk of the disease under dominant and log-additive models (*p* = 0.001). Moreover, rs391957 in *HSPA5* was related to an elevated risk of the disease under recessive and log-additive models (*p* < 0.02). In addition, *DDIT3*-rs697221 was identified to have a significant association with the risk of lung cancer under all three genetic models (*p* < 0.01).

**TABLE 5:** Association between SNPs and risk of lung cancer in genetic models.

SNP	Model	Genotype	Control	Case	OR (95%CI)	*p*
rs6750998	Dominant	TT	335 (54%)	388 (62.6%)	1	0.002*
		TA-AA	285 (46%)	232 (37.4%)	0.70 (0.56–0.88)	
	Recessive	TT-TA	573 (92.4%)	590 (95.2%)	1	0.046*
		AA	47 (7.6%)	30 (4.8%)	0.62 (0.38–1.00)	
	Log-additive	---	---	---	0.74 (0.61–0.89)	0.001*
rs17037621	Dominant	TT	282 (45.5%)	261 (42.1%)	1	0.230
		TA-AA	338 (54.5%)	359 (57.9%)	1.15 (0.92–1.44)	
	Recessive	TT-TA	554 (89.3%)	555 (89.5%)	1	0.930
		AA	66 (10.7%)	65 (10.5%)	0.98 (0.68–1.41)	
	Log-additive	---	---	---	1.08 (0.91–1.28)	0.390
rs867529	Dominant	GG	268 (43.2%)	212 (34.2%)	1	0.001*
		GC-CC	352 (56.8%)	408 (65.8%)	1.48 (1.17–1.86)	
	Recessive	GG-GC	549 (88.5%)	529 (85.3%)	1	0.087
		CC	71 (11.4%)	91 (14.7%)	1.34 (0.96–1.86)	
	Log-additive	---	---	---	1.32 (1.12–1.56)	0.001*
rs17840761	Dominant	GG	222 (35.8%)	202 (32.6%)	1	0.240
		GA-AA	398 (64.2%)	418 (67.4%)	1.15 (0.91–1.46)	
	Recessive	GG-GA	514 (82.9%)	510 (82.3%)	1	0.790
		AA	106 (17.1%)	110 (17.7%)	1.04 (0.78–1.40)	
	Log-additive	---	---	---	1.08 (0.92–1.27)	0.340
rs391957	Dominant	TT	374 (60.3%)	342 (55.2%)	1	0.060
		TC-CC	246 (39.7%)	278 (44.8%)	1.24 (0.99–1.56)	
	Recessive	TT-TC	590 (95.2%)	570 (91.9%)	1	0.017*
		CC	30 (4.8%)	50 (8.1%)	1.76 (1.10–2.81)	
	Log-additive	---	---	---	1.26 (1.05–1.51)	0.014*
rs697221	Dominant	GG	424 (68.4%)	364 (58.7%)	1	0.0004*
		GA-AA	196 (31.6%)	256 (41.3%)	1.53 (1.21–1.93)	
	Recessive	GG-GA	606 (97.7%)	585 (94.3%)	1	0.002*
		AA	14 (2.3%)	35 (5.7%)	2.61 (1.39–4.90)	
	Log-additive	---	---	---	1.52 (1.24–1.86)	<0.0001*

*p < 0.05 indicates statistical significance.

SNP, single-nucleotide polymorphism; OR, odds ratio; CI, confidence interval.

The smoking information was obtained from all the study participants. Therefore, the stratified analysis was performed based on the smoking status (Table 6). We found that the *EIF2AK3*-rs6750998 was a protective factor in both smokers and nonsmokers (*p* < 0.05). In addition, *DDIT3*-rs697221 was still a risk factor in both smokers and nonsmokers (*p* < 0.05). However, *EIF2AK3*-rs867529 and *HSPA5*-rs391957 remained significant only in nonsmokers (*p* < 0.02).

**TABLE 6:** Association between SNPs and risk of lung cancer in smokers and nonsmokers.

SNP	Model	Genotype	Smokers		Nonsmokers	
			OR (95% CI)	*p*	OR (95% CI)	*p*
rs6750998	Dominant	TT	1	0.029*	1	0.035*
		TA-AA	0.72 (0.54–0.97)		0.68 (0.47–0.97)	
	Recessive	TT-TA	1	0.450	1	0.031*
		AA	0.79 (0.42–1.46)		0.45 (0.21–0.95)	
	Log-additive	---	0.78 (0.61–0.99)	0.038*	0.68 (0.51–0.92)	0.010*
rs867529	Dominant	GG	1	0.150	1	0.0003*
		GC-CC	1.24 (0.92–1.66)		1.98 (1.36–2.88)	
	Recessive	GG-GC	1	0.750	1	0.023*
		CC	1.07 (0.70–1.66)		1.83 (1.08–3.09)	
	Log-additive	---	1.14 (0.92–1.42)	0.230	1.66 (1.27–2.18)	0.0002*
rs391957	Dominant	TT	1	0.290	1	0.054
		TC-CC	1.17 (0.87–1.57)		1.44 (0.99–2.09)	
	Recessive	TT-TC	1	0.320	1	0.015*
		CC	1.38 (0.73–2.58)		2.35 (1.15–4.78)	
	Log-additive	---	1.17 (0.92–1.48)	0.210	1.44 (1.08–1.92)	0.012*
rs697221	Dominant	GG	1	0.009*	1	0.011*
		GA-AA	1.48 (1.10–2.00)		1.65 (1.12–2.44)	
	Recessive	GG-GA	1	0.018*	1	0.038*
		AA	2.91 (1.13–7.46)		2.39 (1.02–5.60)	
	Log-additive	---	1.50 (1.15–1.96)	0.002*	1.57 (1.15–2.16)	0.004*

*p < 0.05 indicates statistical significance.

SNP, single-nucleotide polymorphism; OR, odds ratio; CI, confidence interval.

In addition, we also performed a stratification analysis based on the pathological types (Table 7). We found that *EIF2AK3*-rs6750998 was only correlated with a decreased risk of adenocarcinoma (*p* < 0.0016). *EIF2AK3*-rs867529 was associated with an increased risk of adenocarcinoma and squamous cell carcinoma (*p* < 0.025), and *HSPA5*-rs391957 was only related to an elevated risk of squamous cell carcinoma (*p* = 0.0016), while *DDIT3*-rs697221 was associated with risk of all three pathological types (*p* < 0.032).

**TABLE 7:** Association between SNPs and risk of adenocarcinoma, squamous cell carcinoma, and small-cell lung cancer.

SNP	Model	Genotype	Adenocarcinoma		Squamous cell carcinoma		Small cell lung cancer	
			OR (95%CI)	*p*	OR (95%CI)	*p*	OR (95%CI)	*p*
rs6750998	Dominant	TT	1	0.016*	1	0.130	1	0.070
		TA-AA	0.70 (0.53–0.94)		0.72 (0.51–1.02)		0.68 (0.45–1.04)	
	Recessive	TT-TA	1	0.071	1	0.060	1	0.480
		AA	0.57 (0.31–1.08)		0.62 (0.28–1.36)		0.74 (0.32–1.75)	
	Log-additive	---	0.73 (0.57–0.92)	0.008*	0.75 (0.56–1.00)	0.044*	0.74 (0.53–1.05)	0.081
rs867529	Dominant	GG	1	0.003*	1	0.025*	1	0.092
		GC-CC	1.55 (1.15–2.07)		1.48 (1.05–2.09)		1.43 (0.94–2.18)	
	Recessive	GG-GC	1	0.013*	1	0.900	1	0.560
		CC	1.37 (0.91–2.06)		1.03 (0.62–1.73)		1.20 (0.66–2.18)	
	Log-additive	---	1.36 (1.10–1.67)	0.004*	1.24 (0.97–1.59)	0.088	1.26 (0.94–1.69)	0.130
rs391957	Dominant	TT	1	0.093	1	0.430	1	0.150
		TC-CC	1.28 (0.96–1.70)		1.14 (0.82–1.61)		1.35 (0.90–2.03)	
	Recessive	TT-TC	1	0.260	1	0.0016*	1	0.120
		CC	1.41 (0.78–2.54)		2.77 (1.51–5.11)		1.85 (0.87–3.92)	
	Log-additive	---	1.24 (0.98–1.56)	0.070	1.30 (1.00–1.70)	0.054	1.35 (0.98–1.86)	0.073
rs697221	Dominant	GG	1	0.012*	1	0.030*	1	0.015*
		GA-AA	1.46 (1.09–1.96)		1.51 (1.07–2.12)		1.68 (1.11–2.53)	
	Recessive	GG-GA	1	0.032*	1	0.020*	1	0.012*
		AA	2.25 (1.08–4.72)		2.36 (0.95–5.85)		3.45 (1.40–8.50)	
	Log-additive	---	1.45 (1.13–1.86)	0.004*	1.50 (1.11–2.02)	0.009*	1.70 (1.21–2.41)	0.003*

The MDR analysis was further used to evaluate the effect of SNP-SNP interaction on the risk of lung cancer (Table 8). The higher accuracy and cross-validation consistency means a stronger interaction between the SNPs. We found that the interaction model of rs6750998 and rs697221 was the best predictor between candidate genes and lung cancer susceptibility with a testing accuracy of 52%, CVC of 7/10, and *p* < 0.0001.

**TABLE 8:** Summary of SNP-SNP interactions on the risk of lung cancer analyzed by MDR method.

Model	Training accuracy	Testing accuracy	Cross-validation consistency	OR (95%CI)	*p*
rs697221	0.5513	0.5040	5/10	1.521 (1.205–1.920)	0.0004*
rs6750998 and rs697221	0.5598	0.5218	7/10	1.974 (1.500–2.597)	<0.0001*
rs6750998, rs867529, and rs17840761	0.5791	0.4952	3/10	1.842 (1.465–2.316)	<0.0001*

*p < 0.05 indicates statistical significance.

## Discussion

Tumor cells are often in some mal-conditions such as ischemia, low oxygen, and lack of nutrients, resulting in the accumulation of unfolded and misfolded proteins in the ER and causing ER stress (Clarke et al., 2014). ER stress could regulate autophagy, mitochondrial and lysosomal dysfunction, oxidative stress, and inflammatory responses in the tumor, thus playing a vital role in tumorigenesis and tumor metastasis (Lin et al., 2019). In this study, we genotyped six SNPs in ER stress–related genes *EIF2AK3/*PERK, *HSPA5/*GRP78, and *DDIT3/*CHOP in lung cancer patients and healthy individuals and found that *EIF2AK3*-rs6750998 was a protective mutation against the risk of lung cancer, and three SNPs (*EIF2AK3*-rs867529, *HSPA5*-rs391957, and *DDIT3*-rs697221) were risk factors for the disease.

PERK, encoded by *EIF2AK3*, is a type I membrane protein located in the ER and could be activated under ER stress caused by malfolded proteins. The activated PERK could phosphorylate and inactivate the alpha subunit of eukaryotic translation–initiation factor 2 (elF2α), resulting in an effective reduction of translational initiation and repression of protein synthesis (Kranz et al., 2020). In addition, PERK was gradually proved to be involved in the regulation of mitochondrial function, serving as a bridge between mitochondrial metabolism and ER homeostasis (Fan and Simmen, 2019). Küper et al. (2021) have reported that PERK-related phosphorylation of NRF2 is important for the proliferation and ROS elimination of pancreatic and lung cancer cells under constant hypoxia, and thus the PERK-NRF2-HIF-axis contributes to cancer growth. Cai et al. (2021) have found that the PERK-eIF2α-ERK1/2 axis could regulate the cancer-associated fibroblasts to adopt an endothelial cell-like phenotype and directly lead to tumor angiogenesis *in vitro* and *in vivo*. Moreover, Lei et al. (2021) have demonstrated that the PERK activator CCT020312 combined with taxol could significantly reduce the tumor growth in colorectal cancer xenograft, suggesting that promoting PERK might be an effective way to improve colorectal cancer for Taxol treatment. In this study, we identified that two SNPs in *EIF2AK3* were associated with the risk of lung cancer: rs6750998 was a protective SNP against the risk of lung cancer, while rs867529 was a susceptible SNP for the disease. The rs867529 was a missense variant, therefore we speculated that rs867529 may influence the ER stress of the patients with lung cancer by altering the level or function of PERK.

HSPA5 encodes the GRP78 that localizes in the lumen of the ER. GRP78 is a member of the HSP70 chaperone family, making it serve as a molecular chaperone in the folding and assembly of proteins and a regulator of ER homeostasis. Under some conditions that may induce ER stress, such as viral infection and tumorigenesis, GRP78 dissociates from the transmembrane stress sensor proteins PERK, IRE1, and ATF6 and acts as a repressor of the unfolded protein response (Xia et al., 2021). Furthermore, GRP78 also takes part in the process of cellular apoptosis and senescence. Zhang et al. (2021) have shown that GRP78 was upregulated during M2 macrophages polarization, and the downregulation of GRP78 in macrophages suppressed M2 macrophage–provoked proliferation and migration of cancer cells. Huang et al. (2021) have identified that mitochondrial protein ATAD3A could interact with GRP78 to enhance protein folding and reduce ER stress for cancer cell survival in colorectal cancer patients who received chemotherapy. In addition, Gonzalez-Gronow et al. (2021) have reviewed the function studies of GRP78 and concluded that abnormal expression and atypical translocation of GRP78 to the cell surface may be involved in viral infections and pathogenesis of cancers and neurological disorders. Our results have shown that *HSPA5*-rs391957 was related to an elevated risk of lung cancer and rs391957 was a promoter SNP and may lead to altering promoter histone and changed motifs. Therefore, rs391957 may have effects on the risk of the disease due to the altering translocation of GRP78 in lung cancer cells.

CHOP, encoded by *DDIT3*, belongs to the CCAAT/enhancer-binding protein (C/EBP) family. Under ER stress, CHOP was activated by a series of PERK activation and phosphorylation. CHOP could form heterodimers with other C/EBP members to serve as a dominant-negative inhibitor, inhibiting the activity of their binding DNA. Increasing evidence have shown that CHOP was implicated in inflammatory response, poor prognosis, and drug resistance in tumors. Conciatori et al. (2020) have found that the BRAF/ERK2/CHOP axis could regulate the IL-8 transcription *via* regulating the subcellular localization of CHOP and was considered a promising therapeutic target in patients with colorectal cancer. Zhang et al. (2018) have identified that low expression of CHOP was associated with the poor prognosis of patients with advanced gastric cancer, and thus CHOP could be used as a prognostic biomarker for advanced gastric cancer. Xiao et al. (2020) have reported that circRNA_103762 was upregulated in lung cancer tissues, and it could target and inhibit the CHOP expression to enhance the multidrug resistance in lung cancer cells. We identified a missense SNP *DDIT3*-rs697221 that correlated with an elevated risk of lung cancer, suggesting that the minor allele of rs697221 may lead to the dysfunction of CHOP, while the hypothesis needs confirmation through further studies.

Tobacco use is an important risk factor for lung cancer (Raman et al., 2022). We performed a stratified analysis based on smoking status. The results have shown that *EIF2AK3*-rs6750998 was a protective and *DDIT3*-rs697221 remained significant in both smokers and nonsmokers. However, *EIF2AK3*-rs867529 and *HSPA5*-rs391957 were only significant in nonsmokers. The different results may be explained by the limited sample size and other confounding factors such as secondhand smoke exposure, pathological type, and other occupational exposures (de Groot and Munden, 2012). We failed to obtain these information from the participants, which is a main limitation of the present study.

In conclusion, we found that *EIF2AK3*-rs6750998 was a protective variant against the risk of lung cancer, while *EIF2AK3*-rs867529, *HSPA5*-rs391957, and *DDIT3*-rs697221 were all susceptible variants for the disease. These results provided new insights on the role of the ER stress-related gene *EIF2AK3/*PERK, *HSPA5/*GRP78, and *DDIT3/*CHOP polymorphisms for lung cancer risk.

## Data Availability

The original contributions presented in the study are included in the article/Supplementary Material; further inquiries can be directed to the corresponding authors.
